# Graphene damage effects on radiation-resistance and configuration of copper–graphene nanocomposite under irradiation: A molecular dynamics study

**DOI:** 10.1038/srep39391

**Published:** 2016-12-16

**Authors:** Hai Huang, Xiaobin Tang, Feida Chen, Jian Liu, Huan Li, Da Chen

**Affiliations:** 1 Department of Nuclear Science & Engineering, Nanjing University of Aeronautics and Astronautics, Nanjing, 210016, China; 2 Jiangsu Key Laboratory of Nuclear Energy Equipment Materials Engineering, Nanjing, 210016, China

## Abstract

Metal–graphene nanocomposite is a kind of potential radiation tolerant material. Graphene damage of the composite is inevitable within radiation environments. In this paper, two kinds of copper–graphene nanocomposite (CGNC) systems containing perfect graphene and prefabricated damage graphene, respectively, were adopted to expound the influences of graphene damage on the properties (radiation-resistance and configuration) of CGNC under irradiation by atomistic simulations. In the CGNC containing perfect graphene, the increasing graphene damage induced by the increase of the number of cascades, did not obviously impair the role of copper–graphene interface in keeping the properties of CGNC. In the CGNC containing prefabricated damage graphene, the properties of CGNC would significantly deteriorate once the radius of prefabricated damage exceeds 10 Å, and even stacking fault tetrahedral would occur in the CGNC. The results highlighted that prefabricated graphene damage have greater effects on the change of the properties of CGNC. Therefore, it is very necessary to maintain the structural integrity of graphene for improving the radiation-resistance and configuration of CGNC.

Nanostructured metals and composites provide a path for the design of radiation tolerant materials because they contain interfaces that can attract, absorb and annihilate point and line defects^1^,^2^,^3^,^4^,^5^,^6^,^7^. Graphene is well known to be a very flexible and strongest nanomaterial^8^,^9^. If graphene is uniformly dispersed inside the metal matrix, its high aspect ratio and large surface area should create a plentiful of internal metal–graphene interfaces^9^,^10^,^11^,^12^. As a result, the metal–graphene nanocomposite may have the excellent ability to resist radiation damage, and become a candidate as structural material for nuclear applications where high radiation tolerance is a primary concern^13^,^14^,^15^. For example, in the next generation of nuclear reactors, structural materials should be able to endure the much higher neutron doses for long periods of time without failure^13^. In addition, long-term or high dose-rate radiation exposure is also a challenge for the survival of spacecraft^15^.

However, to our knowledge, metal–graphene nanocomposite is still new, and even the most fundamental radiation damage studies, such as point-defect evolution processes, are lacking. In our previous work, a copper–graphene nanocomposite (CGNC) system was adopted and observed under collision cascades by atomistic simulations^16^. Copper was used as the matrix material because it is an ideal metal for fundamental radiation damage studies and has reliable interatomic potentials in MD simulations^2^,^3^,^17^,^18^. The simulation results showed that the surviving defects in the bulk region of CGNC were always less than those of pure copper, thereby implying that CGNC resulting from copper–graphene (Cu–C_gr) interfaces exhibited the excellent radiation resistance^16^. The excellent radiation resistance of V-graphene nanocomposite was also demonstrated by the He^+^ irradiation experiments of Kim *et al*.^19^. However, it was also found that a primary knock-on atom (PKA) with the higher energy corresponded to the worse radiation damage of graphene in CGNC, eventually causing the copper atoms near the damage region of graphene to dissolve and form a columnar block through the region of graphene damage (GD)^16^. Note that PKAs are the atoms directly struck by a high-energy neutron or incident ion in a crystalline material and can launch displacement cascades. Thus, we suspected that the radiation damage of graphene might impair interface stability and eventually weaken radiation resistance of CGNC. But the surmise has not been researched deeply. Moreover, since the graphene of CGNC before irradiation was considered to be perfect, the simulations about the CGNC containing defective graphene before irradiation have not been explored in radiation environments. With the current immature preparation technology of CGNC^10^,^12^,^20^,^21^, it is difficult to obtain the CGNC which contains perfect graphene. For example, the sintering processes could destruct the graphene or graphene oxide (GO) of CGNC since they may involve temperatures higher than the decomposition temperature (~873 K) of graphene or GO^12^. The reduction processes would make the quality control for the graphene of CGNC synthesized by electrochemical deposition difficult and complicated^10^. The CGNC containing defective graphene should be considered. As a result, the defective graphene may have an unusual impact on the radiation-resistance and configuration of CGNC. Therefore, it is necessary to explore the relationships between the graphene damages (irradiated damage and the prefabricated damage introduced before irradiation) and the properties of CGNC (radiation-resistance and configuration).

In this work, two kinds of CGNC systems containing perfect graphene and prefabricated damage graphene, respectively, were adopted to study the effects of GD on the radiation-resistance and configuration of CGNC by the method of molecular dynamics (MD). To better distinguish the damage size (radius R = 0, 5, 10, 15, 20 and 25 Å) of pristine graphene of CGNC, six CGNC models, defined as Cu–Gr/R0, Cu–Gr/R5, Cu–Gr/R10, Cu–Gr/R15, Cu–Gr/R20, and Cu–Gr/R25, were used. As a control, pure copper with a prefabricated copper damage (CD), namely, Cu/R0, Cu/R5, Cu/R10, Cu/R15, Cu/R20, or Cu/R25, was also studied. Full details of these models are discussed in the methods section. For the CGNC system containing perfect graphene (Cu–Gr/R0), the evolution of GD induced by cascade overlaps was investigated firstly. Then, the relationship between the defect production of CGNC and the corresponding GD was revealed. Furthermore, the way that irradiated GD affected the structural change of CGNC, was also discussed. For the CGNC system containing prefabricated damage graphene (Cu–Gr/R0, Cu–Gr/R5, Cu–Gr/R10, Cu–Gr/R15, Cu–Gr/R20, or Cu–Gr/R25), the transformation of prefabricated GD of different sizes before and after irradiation, the corresponding relationship between the defect number of CGNC and the size of prefabricated GD, and the structural change induced by prefabricated GD and irradiation were clarified, respectively. Our results highlighted that the GD of CGNC, introduced by either irradiation or artificiality, played an important role in the radiation-resistance and configuration of CGNC.

## Results and Discussion

### Radiation-resistance of Cu–Gr/R0 after cascade overlaps

**Figure 1:**
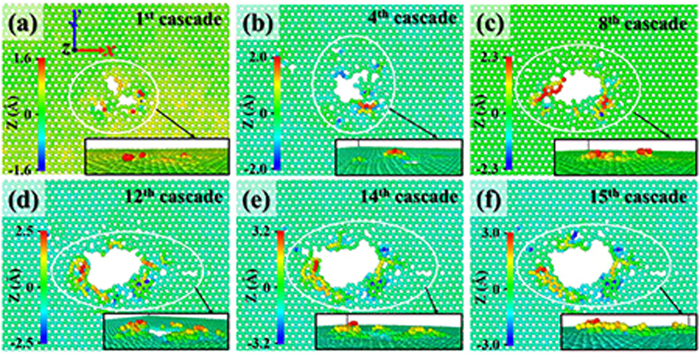
The graphene of Cu–Gr/R0 obtained after the first cascade (**a**), and fourth (**b**), eighth (**c**), twelfth (**d**), fourteenth (**e**), and fifteenth (**f**) overlapped cascades. Carbon atoms are colored according to their z-coordinates centering on graphene layer. The damage region of graphene is singled out with a white oval and enlarged in the insets.

The graphene of Cu–Gr/R0 obtained after the first cascade, and fourth, eighth, twelfth, fourteenth, and fifteenth overlapped cascades is presented in Fig. 1(a–f), respectively. Carbon atoms are colored according to their z-coordinates. It can be seen that with the increase of the number of cascades, GD gradually increases and the motion range of carbon atoms along the z-axis also increases accordingly. The carbon atoms, which are knocked out of the graphene plane due to cascades, cannot escape from the graphene to enter the deep bulk region. On the contrary, they are absorbed on the surface of graphene discretely, or curled up into a flake, thus increasing the thickness of graphene around the edge of GD. The emergence of the above phenomena is attributed to the strong bond energy of C–C, which may bound the motion of carbon atoms, slow the damage rate of GD edges, prolong the irradiated lifetime of the nanocomposite to some degree, and reduce impurity carbon atoms of matrix material. The slight variation between the GD after the fourteenth cascades and the GD after the fifteenth cascades may be affected by the above mechanism, as shown in Fig. 1(e,f).

**Figure 2:**
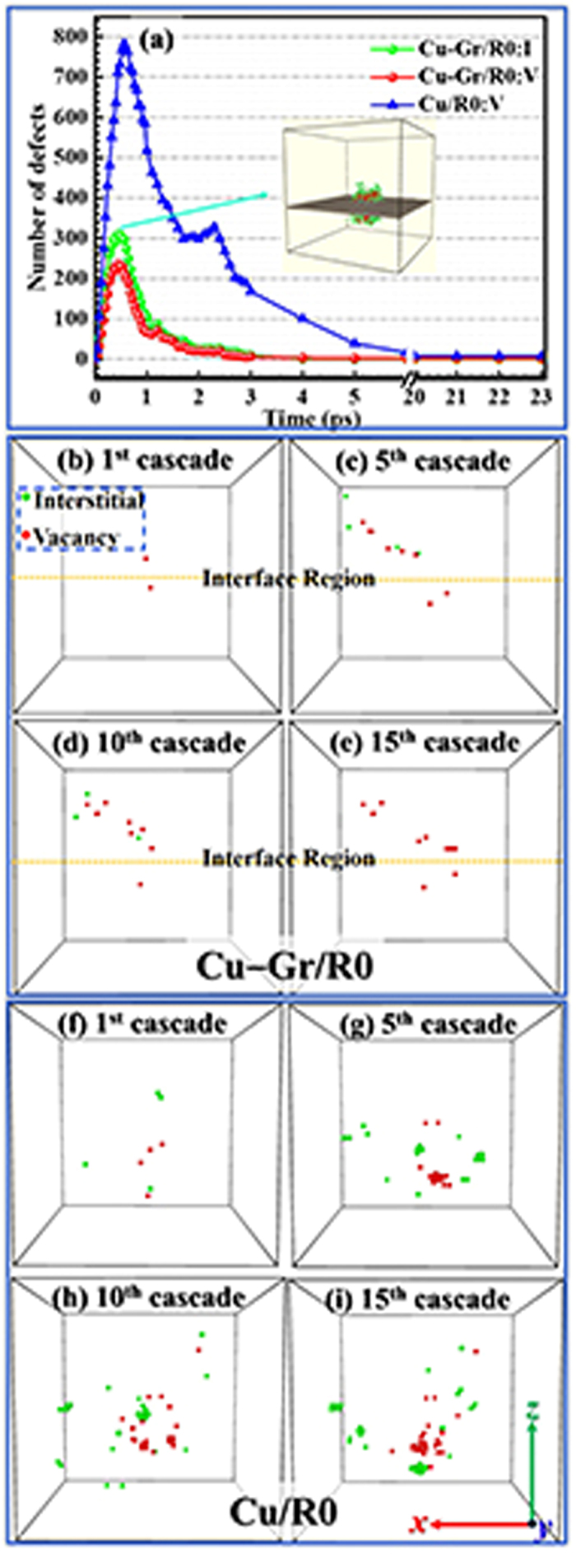
(**a**) The time evolution of the number of interstitials and vacancies produced in the bulk region of Cu–Gr/R0 and Cu/R0 during the first cascade. The defects distribution of Cu–Gr/R0 at damage peak was shown in the inset. The green, red, and gray spheres represent interstitials, vacancies, and carbon atom in the Cu–Gr/R0, respectively. Note that the number of vacancies and interstitials are equal in the Cu/R0, and only the time evolution of vacancies of Cu/R0 is shown. (**b**)–(**i**) The defect distributions obtained after the first cascade, and fifth, tenth, and fifteenth overlapped cascades in the bulk region of Cu–Gr/R0 (panels (a), (b), (c), and (d)) and Cu/R0 (panels (e), (f), (g), and (h)). The green and red spheres represent interstitials and vacancies in the bulk region or pure copper, respectively. The dashed yellow line is to divide the defects at the top and the bottom of the interface.

In order to clarify the role of Cu–C_gr interface in the defect formation or annealing stage, it is appropriate to adopt the results of first cascade because the first cascade is the starting point of fifteen overlapped cascades and can be better to distinguish the evolution of defects. The time evolution of the number of interstitials and vacancies produced in the bulk region during the first cascade was shown in Fig. 2(a). It can be seen that the number of defects increases rapidly at the beginning and reaches a maximum value at approximately 0.5 ps. Eventually, most of defects tend to recombine or are trapped by the Cu–C_gr interface, and very few of them as stable defects are leaved in the bulk region at approximately 23 ps. A similar trend also appears in Cu/R0. It should be noted that the orientation and energy of each PKA as well as the simulation methods of Cu/R0 were consistent with those of the Cu–Gr/R0 simulations and that the similar comparison studies were also simulated for other models. However, the maximum of point defects produced in the bulk region is significantly less than that in the Cu/R0, and the defect concentration in the bulk region is quicker to decrease than that of Cu/R0, which highlight the role of Cu–C_gr interface in inhibiting radiation defects.

Figures 2(b–e) show the snapshots of the defect distributions of bulk region after the first cascade, and fifth, tenth, and fifteenth overlapped cascades at 300 K, respectively. The green and red spheres represent interstitials and vacancies in the cell, respectively. In the four snapshots of Cu–Gr/R0, the number of vacancies shows a tendency of increase and the position of vacancies has little change with the increase of cascade overlaps. The number of interstitials fluctuates obviously and tend to disappear. These phenomena suggests that vacancies are more difficult to migrate at 300 K within typical MD time span. In addition, defects are mainly concentrated in the upper bulk region, implying that the damage of graphene makes kinetic atoms easily penetrate the interface and cause more damage in the upper bulk region than that in the lower bulk region. The defect distributions of Cu/R0 corresponding to those of Cu–Gr/R0 are illustrated in Fig. 2(f,i). In the four snapshots of Cu/R0, the number of defects increases gradually with the increase of the number of cascade overlaps, and most of vacancies are concentrated in the center of the cell while interstitials are mainly distributed in the edge of the cell. The defect distributions of Cu/R0 can be easily explained by the thermal spike phase^22^. Compared the defect distributions of Cu–Gr/R0 with that of Cu/R0, it is easily found that the number of defects in the Cu–Gr/R0 is significantly less than that of Cu/R0. In addition, unlike the Cu/R0, no defect cluster was formed in the Cu–Gr/R0 after the fifteenth overlapped cascades, indicating the role of the interface in suppressing the growth of defects.

**Figure 3:**
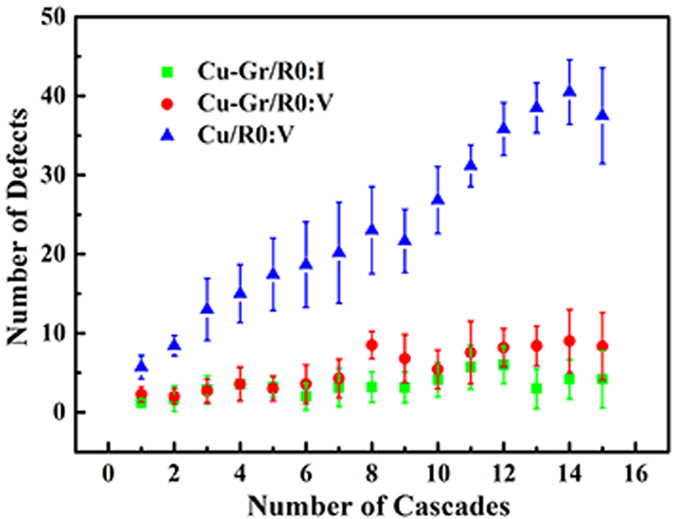
The number of surviving point defects in the bulk region of Cu–Gr/R0 versus the number of cascades. The green box and red circle represent the numbers of interstitials and vacancies in the bulk region, respectively. The number of the vacancies of Cu/R0 is also shown as the blue triangle, while the number of the interstitials of Cu/R0 does not appear due to the equivalence between the interstitials and vacancies in the Cu/R0.

Figure 3 shows the number of surviving point defects (interstitials and vacancies) in the bulk region versus the number of cascade overlaps. Each data point in Fig. 3 (and thereafter) is an averaged value of 10 independent MD runs. The number of surviving point defects shows a slightly rising trend with the increase of the number of cascades. After each cascade, the number of surviving interstitials is less than that of vacancies, indicating that the interface prefers to absorb interstitials and a high concentration of immobile vacancies is left in the bulk region. As shown in Fig. 3, the number of vacancies in the Cu/R0 increases obviously with the increase of the number of cascades. The number of defects (interstitials or vacancies) in the bulk region is always less than that of Cu/R0. The difference of the defect number (especially interstitial number) between the bulk region and Cu/R0 gradually widens with the increase of the number of cascades, further indicating that the Cu–Gr/R0 has the better radiation tolerance compared to its bulk counterpart in the early stage of irradiation. However, combined with Figure 1, it can be seen that bigger GD did not significantly induce the increase of defects. On the contrary, the number of defects may even decrease. For example, the interstitial number after the fourteenth cascades is less than that after the twelfth cascades, as shown in Fig. 3. The above phenomena imply that it is difficult to reveal the relationship between the radiation damage of graphene and the radiation-resistance of Cu–Gr/R0. More cascades are required in our future work.

### Configuration of Cu–Gr/R0 after cascade overlaps

**Figure 4:**
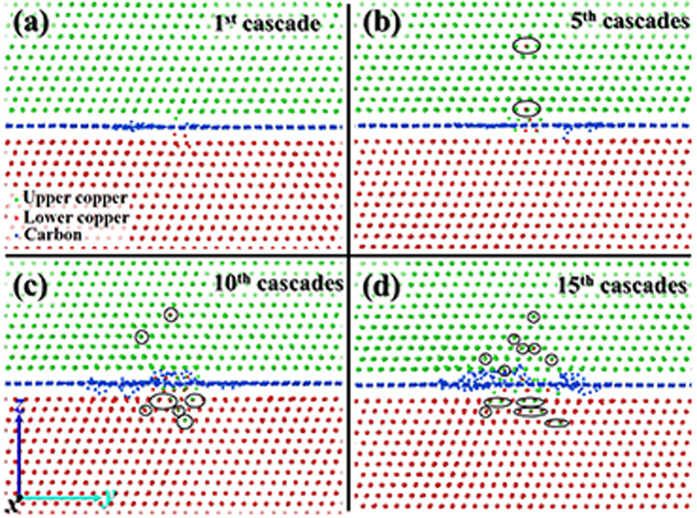
The atoms near the interface obtained after the first cascade (**a**), and fifth (**b**), tenth (**c**), and fifteenth (**d**) overlapped cascades. The displayed area is the cross-sectional view of yz-plane of Cu–Gr/R0. The green, red, and blue spheres represent the copper atoms above the graphene, the copper atoms below the graphene, and the carbon atoms of graphene, respectively. Mutual infiltration between the copper atoms at the top and the bottom of graphene is circled by the black ovals.

With the increase of cascade overlaps, the number of defects in the bulk region slightly increases, and then may lead to the structural change near the Cu–C_gr interface. The atoms near the interface after the first cascade, and fifth, tenth, and fifteenth overlapped cascades are exhibited in Fig. 4(a–d), respectively. With the increase of the number of cascades, the graphene became gradually amorphous in the damage region and carbon atoms were increasingly scattered to the terminal copper layer near the graphene but did not enter the deep bulk region (Fig. 4). The gap between copper and graphene, which may play the role of defect absorption or buffer site, was filled with more and more copper atoms. These copper atoms might be introduced by the knock-on effect or the attraction of the interface. Mutual infiltration as the black oval depicted in Fig. 4, would happen increasingly between the copper atoms at the top and the bottom of graphene due to the increasing size of GD, whereas it did not significantly promote the formation of defects according to the number of defects depicted in Fig. 3. The above phenomena indicate that the increasing size of irradiated GD would not obviously induce structural changes. Therefore, the interface formed by the perfect graphene is very important for the configuration stability of Cu–Gr/R0 during irradiation.

### Radiation-resistance of CGNC of prefabricated GD after single cascades

**Figure 5:**
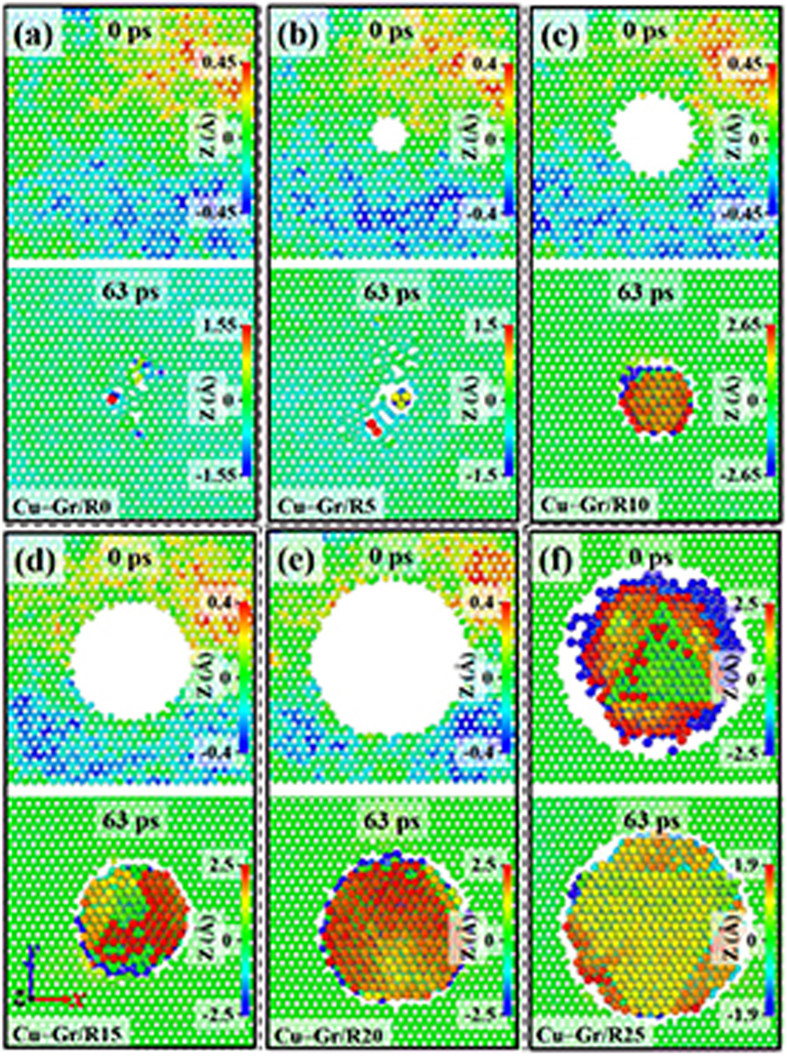
The carbon atoms near prefabricated GD of the six CGNC models and the copper atoms in the gap between copper and graphene. The configurations of each model can be distinguished by the time of 0 ps and 63 ps, respectively. Note that the time of 0 ps and 63 ps represents the starting point and ending point of cascades of each model, respectively. Atoms are colored according to their z-coordinates centering on graphene layer. The spheres with bigger size represent copper atoms, and the rest represents carbon atoms.

The carbon atoms near the prefabricated GD of six CGNC models are shown in Fig. 5(a–f), and the copper atoms in the gap between copper and graphene are also presented to illustrate the miscibility. The configurations of each model can be distinguished by the time of 0 ps and 63 ps, respectively. Note that the time of 0 ps and 63 ps represents the starting point and ending point of cascades of each model, respectively. Atoms are colored according to their z-coordinates, as depicted in Fig. 5. The spheres with bigger size represent copper atoms, and the rest represents carbon atoms. After relaxation, the position of prefabricated GD when R is less than 20 Å, did not appear to copper atoms. Whereas the miscibility between copper atoms occurred in the prefabricated GD of Cu–Gr/R25. In addition, to avoid the neglected miscibility in other models due to the lack of relaxation time, the Cu–Gr/R20 was chosen as a representative to relax at the NVT ensemble for additional 100 ps. The result shows that no miscibility happened in the Cu–Gr/R20, which implies the stability of the CGNC when R is less than 20 Å. After cascades, more copper atoms were stranded near the GD with the increase of prefabricated GD. Due to cascades, the GD would become bigger than that before cascades when R is less than 10 Å, while the GD would be almost unchanged when R is more than 10 Å. The phenomena occurred in six pure copper models were roughly similar to those of CGNC as shown in Supplementary Fig. S4. It should be noted that the distance between a PKA and prefabricated damage layer in pure copper is roughly equal to that of CGNC. However, the most obvious difference between CGNC and pure copper is whether the miscibility of copper atoms near prefabricated damage layer did happen or not, when R is less than 25 Å before cascades. The results imply the role of graphene in retarding the miscibility of copper atoms compared with copper layer.

**Figure 6:**
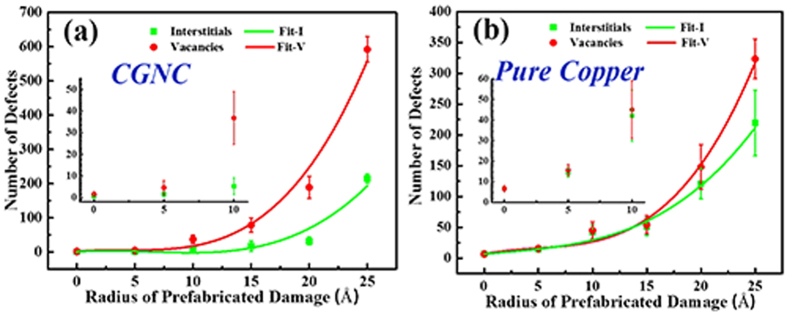
The number of surviving point defects in the bulk region of the six CGNC models or in the six pure copper models as a function of the radius of prefabricated damage. The green box and red circle represent the numbers of interstitials and vacancies in the bulk region or pure copper. The numbers of interstitials and vacancies have been fitted and shown as the green and red lines, respectively. The results of R = 0, 5, and 10 Å are amplified and shown in the inset.

The number of defects in the bulk region versus R value of prefabricated GD after cascades is given in Fig. 6(a). The number of surviving interstitials is always less than that of vacancies, indicating that the interface prefers to absorb interstitials or the stress of cascades induced interstitials to fill prefabricated GD. Both the number of defects and the number difference between vacancies and interstitials gradually increase with the increase of R value. In addition, the number of defects in pure copper versus R value of prefabricated CD after cascades is also given in Fig. 6(b). Compared with the defect number of pure copper, the number of defects in the bulk region of CGNC is much less when R is less than 10 Å, especially interstitials, indicating that the Cu–C_gr interface can act as sinks to facilitate the recombination and annihilation of defects. However, the numbers of vacancies and interstitials, either in the bulk region or in pure copper, rapidly increase when R is greater than 10 Å, and the increase rate of interstitials in the bulk region is slower than that of pure copper while the situation of vacancies is opposite. The results suggest that Cu–C_gr interface still can play an important role in accelerating interstitials or copper atoms to fill prefabricated damage region and leave many vacancies in the bulk region, though the role will be significantly weakened if the size of prefabricated GD exceeds some threshold.

Combined with Figs 5 and 6(a), it is easily found that the ability of graphene of prefabricated damage to curb the miscibility of copper atoms could affect the number of defects in the bulk region. When R is less than 10 Å, the miscibility of copper atoms became weak and the number of defects in the bulk region was very less. When R is more than 10 Å, the drastic miscibility of copper atoms promoted the rapid increase of defects in the bulk region. The above phenomena suggest that the number of defects is affected by irradiation together with prefabricated GD. Therefore, it is necessary to control the size of prefabricated GD for maintaining the role of interface in inhibiting the defects of bulk region. Similar phenomena also occurred in pure copper.

### Configuration of CGNC of prefabricated GD after single cascades

**Figure 7:**
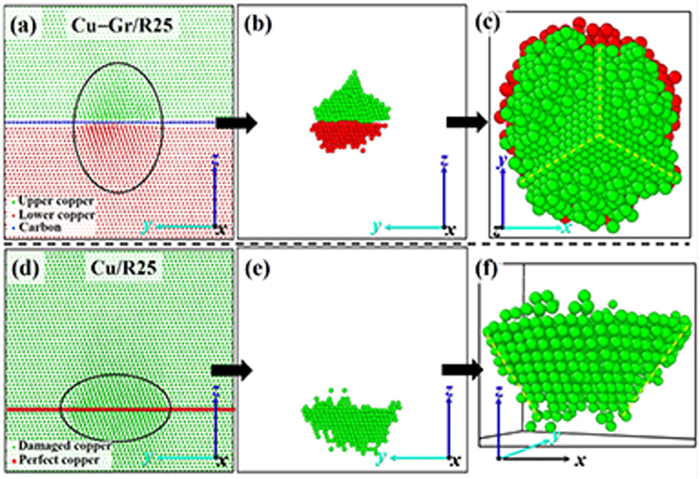
The structures after relaxation corresponding to Cu–Gr/R25 (**a**) and Cu/R25 (**d**), respectively. The deformed regions of Cu–Gr/R25 and Cu/R25 are singled out with a black oval in panels (**a**) and (**d**), and separately shown in panels (**b**) and (**e**). The top-view of panel (**b**) and the vertical view of panel (**e**) are shown in panel (**c**) and panel (**f**), respectively. In the Cu–Gr/R25, the green, red, and blue spheres represent the copper atoms above the graphene, the copper atoms below the graphene, and the carbon atoms of graphene, respectively. In the Cu/R25, the red and green spheres represent the copper atoms on the prefabricated damage layer and the copper atoms of rest region, respectively. For the sake of visualization, the atomic size is not fixed.

Prefabricated GD have great influences on the formation of defects which may disturb the structure of CGNC around the interface. To illustrate the pure effect of prefabricated GD on structural change before cascades, the Cu–Gr/R25 is as an example for analysis. As shown in Fig. 5(f), the miscibility between the copper atoms near the GD of Cu–Gr/R25 would occur. The amorphous structure is presented in Fig. 7(a). The deformed region of copper is picked out from Fig. 7(a), and separately shown in Fig. 7(b). The deformed region includes an embryonic stacking fault tetrahedron (SFT) and an established SFT depicted in Fig. 7(c). For comparison, the structural change of Cu/R25 before cascades is also presented in Fig. 7(d–f). The results indicate that prefabricated damage, either in CGNC or in pure copper, has a great influence on the formation of SFT. However, before cascades, miscibility or SFTs did not occur in other CGNC models, but occurred in each model of pure copper apart from Cu/R0, implying the role of graphene in keeping the stabilization of Cu–C_gr interface.

**Figure 8:**
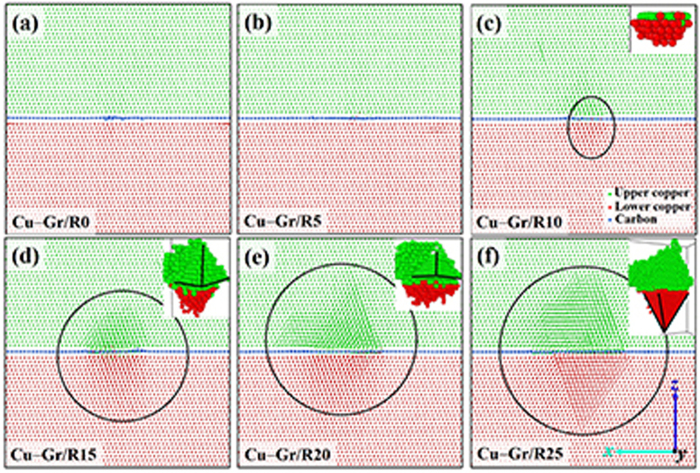
The six CGNC structures after cascades. The SFT region is singled out with a black oval, and separately shown in the inset of panels (c), (d), (e), or (f), respectively. The green, red, and blue spheres represent the copper atoms above the graphene, the copper atoms below the graphene, and the carbon atoms of graphene, respectively.

The six CGNC structures obtained after cascades are shown in Fig. 8(a–f). The structural deformation of CGNC gradually increases with the increase of the size of prefabricated GD. Embryonic SFTs emerge when R trends toward 10 Å, as shown in Fig. 8(c). Two obvious SFTs have been almost completed when R is approximately 25 Å, as shown in Fig. 8(f). Compared Fig. 8(f) with Fig. 8(a), it is easily found that SFTs would rapidly grow due to the facilitation of cascades, indicating the synergistic effects of prefabricated GD and irradiation on the structural change. Considering the effect of prefabricated GD on the number of defects in the bulk region (Fig. 6(a)), we believed that the formation of SFTs near the interface (Fig. 8) was responsible for the increase of the defect number in the bulk region. When R exceeds 10 Å, the sprout of SFTs started to weaken the radiation-resistance of CGNC; while R is less than 10 Å, no SFT occurred and the radiation-resistance ability of CGNC was maintained. Because the prefabricated GD in the graphene layer would induce the appearance of strain in the copper and the irradiation could lead to the formation of voids in the bulk region, it is postulated that the formation of SFTs may derive from the strain together with the voids. Similarly, the larger prefabricated CD exhibits more complicated SFTs after cascades, as depicted in Supplementary Fig. S5. The results highlight the role of graphene in preventing the formation of SFT when R is less than 10 Å. Therefore, apart from the irradiation, the root of SFTs, namely prefabricated GD, should be removed as much as possible for the radiation-resistance and configuration of CGNC.

## Conclusions

Classical molecular dynamics simulations were performed to study the influences of the GD derived from irradiation or artificiality on the radiation-resistance and configuration of CGNC. The influences are summarized as follows.

The results of the CGNC containing perfect graphene by cascade overlaps showed that with the increase of the number of cascades, GD would gradually increase and the number of surviving defects in the bulk region fluctuated slightly, implying that the strict correspondence between the radiation damage of graphene and the radiation-resistance of Cu_Gr/R0 does not exist. Moreover, the defects in the bulk region were always less than that of Cu/R0, demonstrating that Cu_Gr/R0 has better radiation tolerance compared to its bulk counterpart in the early stage of irradiation. Furthermore, mutual infiltration would increasingly happen between the copper atoms at the top and the bottom of graphene, but had little effect on the configuration, indicating the role of perfect graphene in the configuration stability of CGNC.

The results of the CGNC containing prefabricated GD by cascades showed that the radiation-resistance of CGNC can be maintained and the configuration of CGNC was seldom changed when R was less than 10 Å. The defects in bulk region would surge and SFTs emerged once R exceeded 10 Å. The above phenomena highlighted the role of prefabricated GD, which can induce the formation of SFTs and weaken the radiation-resistance and configuration of CGNC. Therefore, the root of SFTs, namely prefabricated GD, should be removed as much as possible.

The CGNC containing perfect graphene may also generate SFTs near the Cu–C_gr interfaces after irradiation. However, within typical MD time span, no SFTs was observed in the CGNC model. The most likely reason may be that deformation stress did not occur in the copper along Cu–C_gr interface. Moreover, the profile of GD is also a key factor for the formation of SFTs and may affect the probability of the occurrence of SFTs. After a long-term irradiation, the perfect graphene of CGNC will be subjected to serious damage and plentiful SFTs may occur. Due to SFTs corresponding to the bad radiation-resistance of the nanocomposite, the observation may be important for evaluating the service lifetime of the nanocomposite.

## Methods

### Interatomic interactions & theoretical models

The interactions among carbon atoms (C–C) in graphene were described by the adaptive intermolecular reactive empirical bond order (AIREBO) potential which has been widely used in the radiation damage of graphene^23^,^24^,^25^. The embedded atom method (EAM) potential was used to describe the interactions between copper atoms (Cu–Cu) splined to the Ziegler-Biersack-Littmark (ZBL) repulsive potential for interatomic distances less than 0.5 Å^2^,^3^,^18^. In describing the interactions between copper and carbon atoms (Cu–C), 12–6 Lennard–Jones (LJ) type of van der Waal’s interaction was used. The LJ potential has been used to simulate cascade collisions of V-graphene nanolayered composite by Kim *et al*.^19^, and the results were approximately consistent with their experimental observations, which proves the feasibility of the potential in cascades simulations of metal-graphene nanocomposite. The parameters (the well depth *σ*_(*Cu-C*)_ = 3.225 Å, equilibrium distance *ε*_(*Cu-C*)_ = 0.019996 eV, and cutoff radius *r*_*c*_ = 2.5*σ*_(*Cu-C*)_) used in this work were derived from Density Functional Theory calculations^26^. Six CGNC structures were generated in this work (Supplementary Figure S1(a)), and the only difference of them is the radius (R) of GD. As shown in Supplementary Fig. S1(b), R values of them are respectively 0, 5, 10, 15, 20, and 25 Å. For the sake of normalization, the R only represents the radius of prefabricated GD before irradiation rather than that of GD induced by radiation in this paper. In order to easily distinguish the six models, different names are defined as: Cu–Gr/R0, Cu–Gr/R5, Cu–Gr/R10, Cu–Gr/R15, Cu–Gr/R20, and Cu–Gr/R25, which correspond to R = 0, 5, 10, 15, 20 and 25 Å, respectively. Similarly, pure copper with a prefabricated copper damage (CD) corresponded to the prefabricated GD of CGNC, was also generated as a control, and is defined as: Cu/R0, Cu/R5, Cu/R10, Cu/R15, Cu/R20, or Cu/R25, respectively. All simulations were performed with the MD code LAMMPS^27^ and visualizations were rendered with OVITO^28^.

### Cascade overlapping simulations in Cu–Gr/R0

To study the relationships between the radiation damage of perfect graphene and the property transitions of CGNC (the transitions of radiation-resistance and configuration) during long-term irradiation, the relaxed Cu–Gr/R0 model was used to simulate cascade overlaps. The simulations were carried out in the following three steps. In Step 1, each cascade was initiated by a PKA with 3.0 keV introduced at a certain distance (*d* = 15.4 Å) from the graphene and directed toward Cu–C_gr interface. The simulation time for each cascade run was 23 ps, which was similar to that of the Fe–Cr alloy^29^,^30^ and enough for the defects to achieve stabilization. In Step 2, the atoms were shifted back over the periodic boundaries to keep the cell centered at the origin after each cascade run in order to allow defect analysis^25^. To quickly release the system stress after the cascade, the nanocomposite containing the defects created by the above cascade was quenched to 0.1 K and then it was re-equilibrated at 300 K after 10 ps. A similar approach was reported in the Fe–Cr alloy under irradiation^29^,^30^. In Step 3, with the relaxed simulation cell of Step 2, Step 1 was repeated for the next cascade (overlap). Up to 15 cascades were simulated in the cell. More detailed simulation settings can be found in the Supplementary Methods.

### Single cascades in six CGNC structures

To study the relationships between the size of prefabricated GD and the properties of CGNC (radiation-resistance and configuration), six relaxed CGNC models were used to simulate collision cascades. The simulation conditions were similar to that of Cu–Gr/R0. At the beginning, a PKA with 3.0 keV in each model was introduced at *d* = 15.4 Å from each graphene and directed toward the center of Cu–C_gr interface, which induced collision cascades subsequently. The choices of energy and position of PKA were similar to that of Cu–Gr/R0. The simulation time for each cascade was set to be 63 ps so that each irradiated model could maintain the stable configuration. In addition, the method of defect analysis and the number of independent simulations were similar to those of Cu–Gr/R0. More detailed simulation settings can be found in the Supplementary Methods.

## Additional Information

**How to cite this article**: Huang, H. *et al*. Graphene damage effects on radiation-resistance and configuration of copper-graphene nanocomposite under irradiation: A molecular dynamics study. *Sci. Rep.* **6**, 39391; doi: 10.1038/srep39391 (2016).

**Publisher’s note:** Springer Nature remains neutral with regard to jurisdictional claims in published maps and institutional affiliations.

## Supplementary Material

Supplementary Information
