# Supplementary material for: Graphene damage effects on radiation-resistance and configuration of copper–graphene nanocomposite under irradiation: A molecular dynamics study
Source: Sci Rep. 2016 Dec 16;6:39391. doi: 10.1038/srep39391 (PMC5159848; doi:10.1038/srep39391)
Supplement: Supplementary Information [file srep39391-s1.pdf]

Supplementary Information

Graphene damage effects on radiation-resistance and configuration of copper–graphene nanocomposite under irradiation: A molecular dynamics study

Hai Huang[1], Xiaobin Tang[1,2,*] , Feida Chen[1], Jian Liu[1], Huan Li[1], and Da Chen[1,2]

[1] Department of Nuclear Science & Engineering, Nanjing University of Aeronautics and Astronautics, Nanjing, 210016, China. [2] Jiangsu Key Laboratory of Nuclear Energy Equipment Materials Engineering, Nanjing, 210016, China.

[*]Corresponding author. Tel.: +86 13601582233; fax: +86 025 52112908-80407.

E-mail address: tangxiaobin@nuaa.edu.cn (X. Tang).

Supplementary Methods: Further descriptions of simulation model and methodology.

Six CGNC structures were generated in this work, and the detail about graphene matching with copper can been found in our previous study[1]. For different CGNC structures, each layer thickness of copper is approximately 56 Å, and both of the crystallographic orientations of copper are x [$\bar{1}$ 1 0], y [$\bar{1}$ $\bar{1}$ 2], z [1 1 1], as shown in Fig. S1(a). The generated details of prefabricated GD in the CGNC structures are described as follows. The center of perfect graphene (R = 0 Å) is regarded as the center of a circle. A certain value is selected as the radius of the circle and all atoms in the circle are deleted, as shown in Fig. S1(b). As an example, the graphene of R = 10 Å is shown in Fig. S1(c). Note that the ring-like defect as the prefabricated damage of graphene were adopted due to the simplicity of the hole-model. In spite of the damage type that is infrequent for graphene, it is an effective approach to approximatively predict the effect of prefabricated GD on the radiation-resistance and configuration of CGNC. The basic information of the six CGNC structures is summarized in Table S1.

To release the stress out of these systems, the minimum energy configurations of the six structures were obtained by applying the conjugate gradient minimization method under zero external pressure[2]. In addition, considering the relatively small number of atoms used in the simulations, the system was simulated by using periodic boundary conditions (PBC) along the three Cartesian directions. The definition methods of interfacial region and bulk region, the purpose of which is to easily distinguish radiation damage, are similar to those in our previous work[1]. According to the visible difference of potential energy shown in Fig. S2(a), the interfacial width can be obtained easily. In each CGNC, interfacial region contains one graphene plane and two terminal copper planes near the graphene, and the rest belongs to the bulk region. As an example, the distributions of the potential energy of different atoms in the Cu–Gr/R10 are shown in Fig. S2(b). Before cascades, all models were relaxed at the NVT ensemble (constant number of atoms, volume, and temperature) with the time step of 10^{-3} ps for 10 ps until each system reached a stable state. The temperature was set to 300 K in this process.

During each cascade, the atoms within the "thermostat region" were forced to maintain a constant temperature (300 K) through the Nose–Hoover heat bath, so that the excess kinetic energy introduced by a PKA could be dissipated as that in experimental situations. The atoms within the "active region" were restricted to move adiabatically (the NVE ensemble), as shown in Fig. S3. Cascade crossing through the cell borders was avoided as much as possible. Each PKA, which could increase the size of GD and maintain cascades in the "active region" as much as possible, was selected in a small central area of the copper plane 15.4 Å from the graphene. The choices of energy and position of PKA were based on these two points. Firstly, the energy of PAK has been picked usually in the range of 1 keV to 10 keV for the study of radiation damage[2-6]. In this work, the PKA with 3.0 keV which corresponds to the maximum transferred

by an elastic collision of 0.05 MeV neutron, was appropriate to study the role of the interface as that in Cu-Nb[4]. Secondly, after a lot of tests, the optimum *d* was found to be near the 15.4 Å, in order to achieve displacement peak symmetrical about the Cu–C_gr interface and concentrate defects near the interface with the 3.0 keV PKA. In addition, to reduce the statistical error, 10 independent cascade overlapping simulations were performed. After each cascade, the reference lattice site method with a cutoff distance of 0.3a_0 (a_0 is the lattice constant of copper) was used to characterize the defects in the bulk region[3,4].

Supplementary Table S1. The six CGNC models used in this work.

Types	N_Cu	N_C	A_d/A_r (%)	W (Å)
Cu–Gr/R0	126360	5022	0	6.19076
Cu–Gr/R5	126360	4998	0.6	6.17014
Cu–Gr/R10	126360	4898	2.4	6.16470
Cu–Gr/R15	126360	4748	5.4	6.16484
Cu–Gr/R20	126360	4537	9.6	6.17201
Cu–Gr/R25	126360	4266	15	6.16128

Note: N_Cu is the number of copper atoms; N_C is the number of carbon atoms; A_d/A_r is the ratio of prefabricated damage area to the remaining area of graphene; W is the interfacial width.

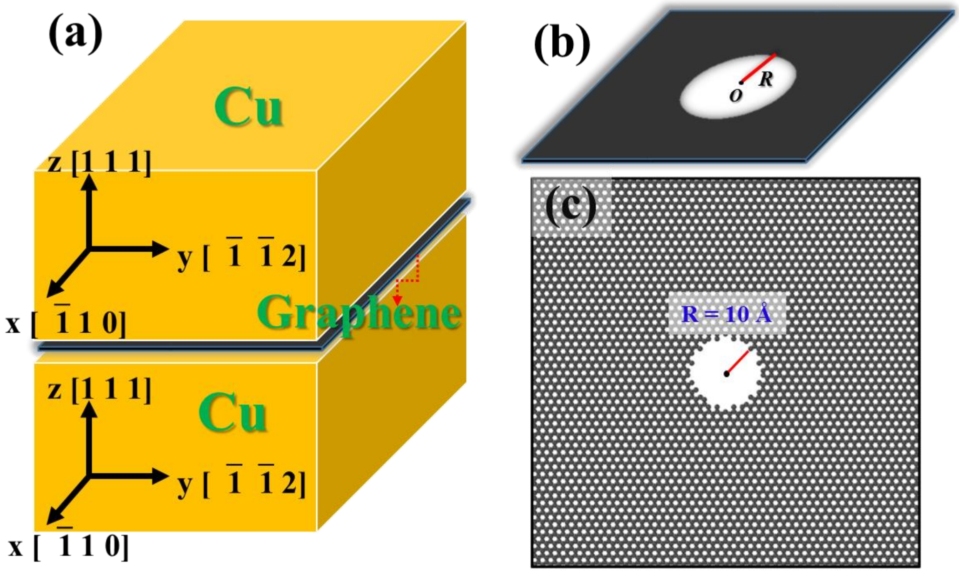

Supplementary Figure S1. (a) A conceptual schematic diagram of CGNC used as simulation cell in this work; (b) the graphene model added into copper and a prefabricated GD exhibited at the center of graphene, in which R values of them are 0, 5, 10, 15, 20, and 25 Å, respectively; (c) the graphene containing a prefabricated GD of R = 10 Å.

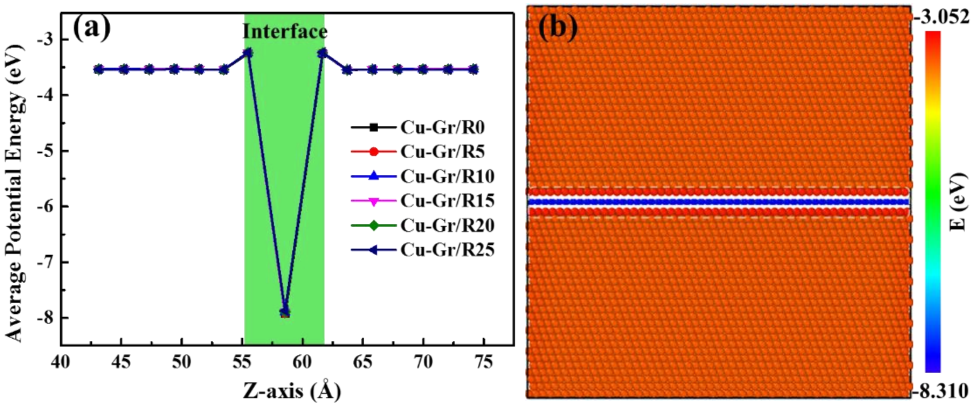

Supplementary Figure S2. (a) Average potential energy of atoms near the Cu–C_gr interface parallel to the interface as a function of the coordinate perpendicular to the interface in the six CGNC structures; (b) the distributions of the potential energy of different atoms in the Cu–Gr/R10 visualized by the atoms colored according to their potential energy. The obviously different potential energy in the center of the system represents the Cu–C_gr interface which contains one graphene plane and two terminal copper planes near the graphene.

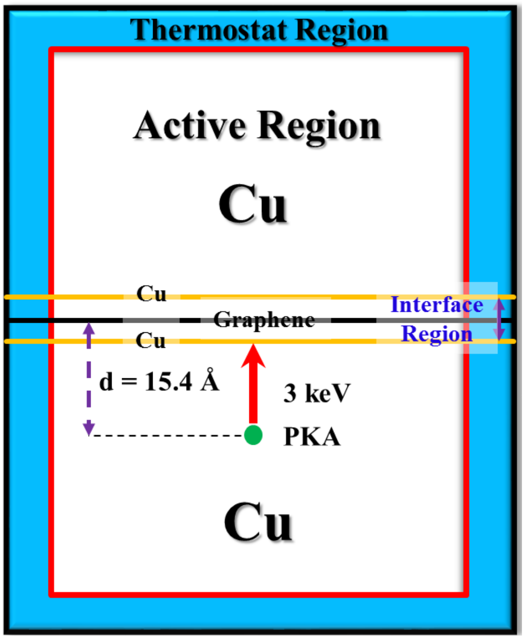

Supplementary Figure S3. The MD configuration viewed in the x-direction. The atoms within the "thermostat region" (along the three directions) are controlled by the NVT thermostat to

maintain a constant temperature. Other atoms (within the ‘‘active region’’) are allowed to move adiabatically as the NVE ensemble. In the simulation cell, the three directions exhibit PBC, and a PKA with 3 keV is at a distance (d = 15.4 Å) away from the graphene.

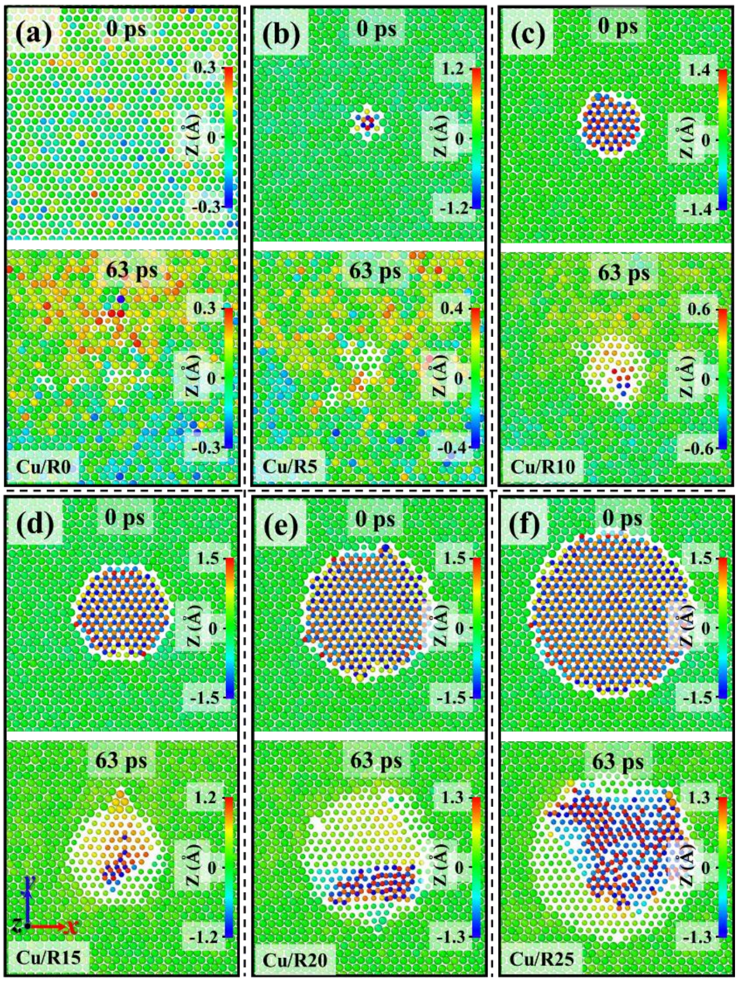

Supplementary Figure S4. The copper atoms near prefabricated damage layer of the six pure copper models. The configurations of each model can be distinguished by the time of 0 ps and 63 ps, respectively. Note that the time of 0 ps and 63 ps represents the starting point and ending point of cascades of each model, respectively. Atoms are colored according to their z-coordinates centering on prefabricated damage layer. The spheres with bigger size represent copper atoms in prefabricated damage layer, and the spheres with smaller size represents the rest atoms.

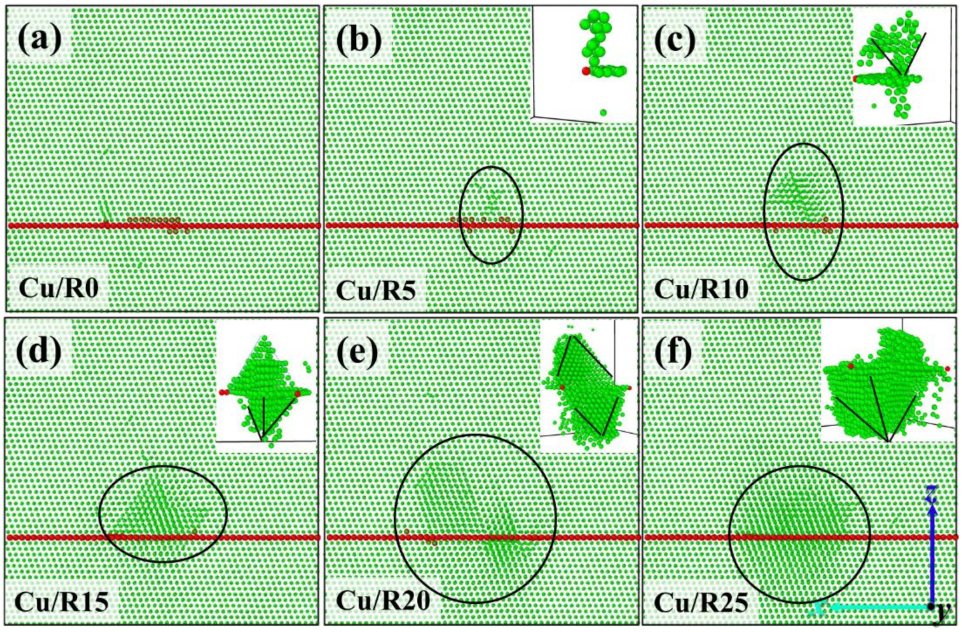

Supplementary Figure S5. The six pure copper structures after cascades. The SFT region is singled out with a black oval, and separately shown in the insets of panels (b), (c), (d), (e), or (f), respectively. The red and green spheres represent the copper atoms on the prefabricated damage layer and the copper atoms of rest region.

Supplementary References

1. Huang, H. *et al*. Radiation damage resistance and interface stability of copper–graphene nanolayered composite. *J. Nucl. Mater*. **460**, 16–22 (2015).
2. Zhang, C., Zhou, W., Li, Y., Zeng, Z. & Ju, X. Primary radiation damage near grain boundary in bcc tungsten by molecular dynamics simulations. J. Nucl. Mater. 458, 138–145 (2015).
3. Bai, X., Voter, A. F., Hoagland, R. G., Nastasi, M. & Uberuaga, B. P. Efficient annealing of radiation damage near grain boundaries via interstitial emission. *Science* **327**, 1631–1634 (2010).
4. Liu, X. *et al*. Mechanism for recombination of radiation-induced point defects at interphase boundaries. *Phys. Rev. B* **85**, 012103 (2012).
5. Diaz de la Rubia, T., Averback, R. S., Hsieh, H. & Benedek, R. Molecular dynamics simulation of displacement cascades in Cu and Ni: Thermal spike behavior. *J. Mater. Res*. **4**, 579–586 (1989).
6. Dai, Y. *et al*. Nucleation of Cr precipitates in Fe–Cr alloy under irradiation. *Comp. Mater. Sci*. **101**, 293-300 (2015).
